# Childhood adversities and distress - The role of resilience in a representative sample

**DOI:** 10.1371/journal.pone.0173826

**Published:** 2017-03-15

**Authors:** Manfred E. Beutel, Ana N. Tibubos, Eva M. Klein, Gabriele Schmutzer, Iris Reiner, Rüya-Daniela Kocalevent, Elmar Brähler

**Affiliations:** 1 Department of Psychosomatic Medicine and Psychotherapy, University Medical Center of the Johannes Gutenberg University Mainz, Mainz, Germany; 2 Department of Medical Psychology and Medical Sociology, University of Leipzig, Leipzig, Germany; 3 Department of Medical Psychology, University Hospital Hamburg-Eppendorf, Hamburg, Germany; Central Institute of Mental Health, GERMANY

## Abstract

While adverse childhood experiences have been shown to contribute to adverse health outcomes in adulthood, specifically distress and somatic symptoms, few studies have examined their joint effects with resilient coping style on adult adjustment. Hence, we aim to determine the association between resilient coping and distress in participants with and without reported childhood adversities. A representative German community sample (*N* = 2508) between 14–92 years (1334 women; 1174 men) was examined by the short form of the Childhood Trauma Questionnaire, the Brief Resilience Coping Scale, standardized scales of distress and somatoform symptoms. Childhood adversity was associated with reduced adjustment, social support and resilience. It was also strongly associated with increased distress and somatoform complaints. Resilient coping was not only associated with lower distress, it also buffered the effects of childhood adversity on distress. Our study corroborates the buffering effect of resilience in a representative German sample. High trait resilient subjects show less distress and somatoform symptoms despite reported childhood adversities in comparison to those with low resilient coping abilities.

## Introduction

Childhood maltreatment has been increasingly recognized as a major public health problem in high-income countries [1–6]. Adverse childhood experiences comprise acts of commission of sexual, physical, emotional abuse as well as acts of omission such as emotional and physical neglect and witnessing intimate partner violence [1, 7]. The experience of being harmed by persons who should provide support and protection leads to severe neurobiological, somatic and mental damage in the developing child, compromising the ability to cope with somatic and psychic stressors throughout lifespan [8]. In numerous studies, adverse childhood experiences have been associated to multiple adverse somatic and mental diseases in adulthood, maladjustment and an unhealthy life style [1, 9–11]. Consequences of adverse experiences also depend on the developmental phase of the individual; most serious consequences are expected from adverse childhood experiences during formative early childhood periods of biological and psychological development when available strategies of coping or defense are still limited [2, 12]. Negative effects can be compounded by genetic risk factors [12, 13], lack of social support [14] and additional adversities (e.g. unemployment) later in life [15].

Yet, not all individuals affected by stressful life events, such as childhood adversities, suffer from psychological distress, such as post-traumatic stress syndromes, or medical disorders later in their lives [16]. In the well-known longitudinal study following the development of nearly 700 subjects on the Hawaiian island of Kauai [17], about a third grew up under high risk conditions such as poverty, parental divorce or mental illness. However, as they became competent and well-adjusted adults, 72 of these high risk children were termed “resilient” towards those risk factors. Werner identified several protective factors related to the successful adaptation in high risk children, e.g. supportive adults and approach-oriented temperamental characteristics.

The concept of resilience has roots in biomedical and psychological disciplines, particularly in developmental psychology. A body of evidence has demonstrated the beneficial effect of resilience over the decades, e.g. [18–22]. Within the field of psychology, there is a variety of conceptual definitions of resilience, e.g. [23–25]. Resilience can be defined as an outcome in the face of adversity or as a process mediating the response to stress or trauma [26]. Resilience factors are empirically derived variables which statistically predict a resilient outcome. Thus, they link two elements, the exposure to risk or hardship and a positive outcome within or higher than the expected range [27]. The most prominent factor is an individual’s ability to respond positively to physiological, psychological or social challenges in the environment [28–30]. Some even thrive under hardships, extracting positive aspects or surpassing earlier functioning after handling stressful life events [31]. These kinds of positive adjustments after adverse life events are defined as steeling [32–34] in a non-trauma related or post-traumatic growth [35] in a trauma related context.

Assessing individual resilience expands our understanding of stress resistance and adaptation. Increasingly, empirical studies focused on identifying the characteristics of individuals, in particular young people, who managed to thrive despite living in difficult circumstances, such as parental mental illness or poverty [36–38]. Resilient individuals have been shown to use effective, active problem-solving patterns [39] and adaptive appraisal styles in terms of coping mechanisms [40]. Thus, resilient coping enables positive adaptation despite extremely stressful circumstances [31].

The Isle of Wight study prospectively assessed psychiatric disorder, peer relationships and family functioning in adolescence and lifetime psychiatric history, personality and social functioning in adulthood along with retrospective adult reports of childhood sexual and physical abuse [41]. According to Collishaw et al. [42], ten percent of individuals reported repeated or severe physical or sexual abuse in childhood. Prospective measures revealed increased rates of adolescent psychiatric disorders and high rates of adult psychopathology in this group. A substantial minority of about one third of physically or sexually abused individuals, however, reported no mental health problems in adult life. Resilience was related to perceived parental care, adolescent peer relationships, the quality of adult love relationships, and personality style (e.g. low neuroticism). While adverse childhood experiences and resilience have found a renewed interest over the past years, fewer studies have set out to determine the joint effects of adverse childhood experience and resilience, in terms of an interaction effect, on adult health outcomes.

Some studies have demonstrated the moderating effect of resilience as personality trait [22, 43, 44], yet only a few of them have referred to childhood adversities and psychiatric outcomes at the same time [45–47]. However, epidemiological studies which are hardly affected by sample biases due to the underlying method of data collection are scarce in this field (cf. [48]). With regard to the buffering effect of resilience, we found only one cross-sectional study which has addressed the interaction effect of trait resilience and childhood abuse on depression, focused on an urban sample of predominantly African Americans [49]. Therefore, we aim to investigate the buffering effect of dispositional resilient coping style on the link of childhood adversities with mental health based on a representative national data set in a European country and with no restriction to specific subgroups. Additionally, we focus on both, psychological and somatic indicators of health outcomes, in our study. To our knowledge, the current study is the first testing this hypothesis in a representative community sample allowing more valid conclusions due to the higher generalizability of the results.

In a representative community survey, we wanted to answer the following questions:

1. How are childhood adversities associated to adjustment, social support and resilience?
2. What are the effects of childhood adversities and resilience on distress and somatic symptoms?

Our main hypotheses were:

Childhood adversity is associated with unfavorable adjustment, reduced social support and resilience over the lifespan.
a) Childhood adversity is associated with distress and somatic symptoms in adulthood b) This effect is buffered by resilience.

## Method

### Participants

The present study was based on a representative survey of the German population. Data were collected by USUMA (Unabhängiger Service für Umfragen, Methoden und Analysen; Berlin) in June and July 2013. A total of 2,508 participants (1,334 women; 1,174 men) were included between the ages of 14 and 92 years (*M* = 49.7, *SD* = 18.3). For data analysis, the final sample comprised 2,486 participants due to the exclusion of subjects with missing data regarding childhood adversity (S1 Data). As in our previous surveys [7, 50, 51], participants were recruited at 258 sample points of the Eastern and Western states of Germany; the majority (79.9%) lived in the Western states of Germany. Those, who gave informed consent, were interviewed face-to-face by trained staff in their homes and independently filled out additional questionnaires in their presence. Study participants received no incentives. The survey followed ADM (Arbeitskreis Deutscher Markt- und Sozialforschungsinstitute e.V.) sampling guidelines for generating a representative sample of the German population [52]. Sampling was performed in three steps: 1) Areas were regionally stratified for identifying sampling points, 2) private households were selected, 3) the individual within the selected household was determined. The region, the households and target persons were randomly selected by random route procedure. 55.1% of the initial sample (4,607 households) were interviewed, matching quota of other representative population samples. Participants were in the age range of 14 to 92 years. 46.8% were male. 46.1% of the sample was married and 58.1% lived in a partnership. With regard to education, the great majority had completed high school or 10^th^ grade of education (59%). The full or part-time employment rate was 51.2%, the unemployment rate 5.7%, 1.9% worked on a fee-per-hour basis, 4.1% were in charge of the household, 29.7% received pension, 6.0% attended school, 1.7% had vocational training and 0.8% were on parental leave, military or civilian service.

The institutional ethics review board of the University of Leipzig approved the study and procedure (Az 063-14-10032014). The ethics committee of the University of Leipzig approved the consent procedure for the whole sample including participants between 14 and 18 years. Adhered to ICH-GCP-guidelines (ICH = International Conference on Harmonisation of Technical Requirements for Registration of Pharmaceuticals for Human Use; GCP = Good Clinical Practice) and to the guidelines of the ICC/ESOMAR International Code of Marketing and Social Research Practice (ICC = International Chamber of Commerce; ESOMAR = European Society for Opinion and Market Research), all participants were informed of the study procedures, data collection and anonymization of all personal data. Moreover, they were delivered a detailed data privacy statement. The present study posed a low risk to the participants, as medical treatments, invasive diagnostics or procedures causing psychological, spiritual or social harm were not included. Verbal informed consent was given by all participants and was noted by the trained interviewer before starting with the survey. The additional informed consent of a parent was thus not required for participants aged 14 or older.

### Measures

We included living in a partnership, education, total household income and experienced unemployment as determinants of adult adjustment in addition to age (≥14 years) and sex. One established scale for assessing major dimensions of childhood adversity is the short form of the childhood trauma scale (Childhood Trauma Screener, CTS; [9]. The Brief Resilience Coping Scale (BRCS; [31] addresses the flexible use of creative, active problem solving abilities. These include cognitive assessment, mastering difficult situations, compensating losses, and controlling one’s reactions. This coping pattern is based on tenacity, optimism, active problem solving and active extraction of positive growth. We used distress and somatic symptoms as measures for adverse health outcome since they are considered frequent adult sequelae of childhood adversity [53, 54]. Socio-demographic variables were assessed: education, partnership, household income and employment. Perceived social support was measured by the German Social Support Questionnaire (FSozU-6; [55]).

The Childhood Trauma Screener (CTS) is the German short form of the Childhood Trauma Questionnaire (CTQ) with five items [9]. The CTS was used to assess childhood adversity. Participants rated emotional, physical, and sexual abuse as well as emotional and physical neglect on a 5-point scale (1 = “not at all” to 5 = “very frequently”). Despite its brevity, it is a reliable scale (Cronbach´s alpha = .76). Correlations of the five single items (see Table 2) with the 5 respective scales are in a range of r = .55 to .87. Based on a validation study [56], low childhood adversity was defined by CTS scores of 0–10 and high >10, respectively. Childhood adversity was dichotomized according to high (CTS >10) vs. low.

In the Brief Resilience Coping Scale (BRCS) respondents were instructed to rate how well each of four statements describe their behavior and actions (“I look for creative ways to alter difficult situations”, “regardless of what happens to me, I believe I can control my reaction to it”, “I believe I can grow in positive ways by dealing with difficult situations”, “I actively look for ways to replace the losses I encounter in life”). Items are answered on 5 point Likert scales (1 = “not at all” to 5 = “very”). Sinclair and Wallston (31) demonstrated good reliability (Cronbach´s alpha = .69); test-retest correlations were between .71 and .68 [27, 31]. The scale score of the BRCS was transformed into values from 0 to 100 based on the formula BRCS100 = ((((BRCS01 + BRCS02 + BRCS03 + BRCS04) / 4)– 1) / 4) x 100. This transformation procedure is based on pilot studies of the BRCS in the Medical Department Charité Berlin, Division of Psychosomatic Medicine, in which participants received personalized feedback via personal digital assistants. The BRCS score was dichotomized according to median split (>/ < 69). The BRCS has been positively associated with work satisfaction in physicians [57] and negatively with chronic pain [31]. A recent Spanish validation study found positive correlations with personal perceived competence, optimism, life satisfaction and positive affect as well as negative correlations with depression, anxiety and negative affect [58].

The PHQ-4 [59]is an ultra-brief and reliable (Cronbach’s alpha = .80) screener with two factors, depression and anxiety. Depression items assess depressed mood and loss of interest (PHQ-2). Anxiety includes the two screening items of the short form of the GAD-7 (Generalized Anxiety Disorder [GAD]-2 Scale): “Feeling nervous, anxious or on edge” and “not being able to stop or control worrying”. Occurrence in the past two weeks was rated from 0 = “not at all”, 1 = “several days”, 2 = “over half the days”, and 3 = “nearly every day”. PHQ-4 scores are strongly associated with multiple domains of functioning, e.g. work disability).

To assess somatic symptom strain, we used the short form of the Giessen Subjective Complaints List (GBB-8; Gießener Beschwerdebogen [60]). This inventory comprises eight items: easily exhausted, tired/fatigue, pressure in abdomen or abdominal bloating, stomach ache/ abdominal pain, lumbal or back pain, neck or shoulder pain, unpleasant heart beats/tachycardia or arrhythmia, dizziness. Each symptom is rated on a Likert scale from 1 (never) to 5 (always). GBB-8 sum scores range from 8 to 40, whereas higher values indicate higher somatic burden. In the current study, the GBB-8 reached a high internal consistency, Cronbach's alpha = 0.89.

In order to measure perceived social support, we administered the six-item short form of the German Social Support Questionnaire (FSozU-6 [55]). It is a valid and reliable instrument with a 5-point-Likert-scale ranging from 1 = “it does not fit at all” to 5 = “it fits exactly”. Thus, a higher score indicates higher perceived social support. The internal consistency in our sample was very good, Cronbach's alpha = .90.

### Statistical analysis

All data were analyzed by SPSS (Version 23) by univariate and multivariate procedures, using two-sided t-tests and Chi^2^, respectively. In order to maintain representativity and with regard to the neglectable amount of missing data in our main variables which was far below 5% (cf. [61, 62]), we did not impute missing values. For childhood adversities, 22 cases (0.9%) were missing at scale level; at item level a maximum of 14 responses per item were observed. Statistical effects of childhood adversity and resilience on distress were computed by two-way ANOVA with interactions. In multivariate models we identified determinants of resilience and of distress adjusting for socio-demographic variables and adult life stress. Level of significance was set at p < .05, two-tailed.

## Results

### Association of childhood adversity to adult adjustment, social support and resilience

Table 1 shows sample characteristics according to the presence or absence of significant childhood adversity. Significant childhood adversity (CTS >10) was reported by 16.1% of the sample. There were no sex differences. Participants with adverse childhood experience were less likely to live in a partnership and to have achieved high school education. They had a lower income, and they had more frequently experienced unemployment in their lives. Adverse childhood experiences were associated with higher age, reduced social support and low resilience. Table 2 displays gender and age specific statistics. A significant gender difference was observed for sexual abuse. Significant age related differences could be found for physical abuse and both neglect facets. Twice as many female as male participants reported sexual abuse experiences during childhood. Among older participants (> 40 years), physical abuse reports were more frequently. Physical neglect was more frequently reported than emotional neglect. Participants between 31–40 years and those above 60 years reported the highest scores referring to physical neglect during childhood. Emotional neglect was reported twice as often among individuals older than 30 years in comparison to those younger than 30 years.

**Table 1:** Demographic characteristics, perceived social support, and resilience in participants with and without significant childhood adversity.

		Significant childhood adversity in %			
		Total(*N* = 2486)	Yes(*n* = 402)	No(*n* = 2084)	*Chi*^*2*^
**Sex**	female	53.3	55.2	53.0	n.s.
**Partnership**	yes	52.4	47.5	53.5	4.77 *
**Education**	>10th grade	18.2	10.8	19.6	17.66 ***
**Income**	>2000 Euro	48.5	34.9	51.1	33.78 ***
**Experienced unemployment**^1^	yes	41.2	53.9	38.8	31.52 ***
		Mean (SD)	Mean (SD)	Mean (SD)	*t*
**Age**	years	49.67 (18.32)	52.48 (17.41)	49.14 (18.45)	-3.48***
**Social support**		4.01 (0,76)	3.47 (.80)	4.12 (.70)	15.12***
**Resilience**		67.14 (19,9)	13.34 (3.36)	15.02 (3.07)	9.24***

Note:

^1^refers to the entire lifespan.

***p < .001;

*p < .05.

**Table 2:** Gender and age specific descriptive and inference statistics of the childhood adversity items.

total (N = 2486)	*number (percentage) of participants reporting childhood adversity experiences*				
	physical abuse	emotional abuse	sexual abuse	emotional neglect	physical neglect
**female (n = 1326)**	147 (11.0%)	147 (11.0%)	171 (12.8%)	107 (8.0%)	183 (13.8%)
**male (n = 1160)**	146 (12.4%)	103 (8.8%)	65 (5.5%)	95 (8.1%)	131 (11.2%)
**p-value (Kolmogorov-Smirnov-Z)**	1.00	.92	<.01	1.00	.82
**age range (years)**					
**14–30 (n = 472)**	42 (8.8%)	4.2 (8.8%)	42 (8.8%)	21 (4.4%)	33 (6.9%)
**31–40 (n = 337)**	28 (8.3%)	31 (9.1%)	30 (8.8%)	29 (8.6%)	48 (14.2%)
**41–50 (n = 449)**	57 (12.5%)	64 (14.0%)	58 (12.7%)	38 (8.3%)	48 (10.5%)
**51–60 (n = 447)**	73 (16.2%)	50 (11.1%)	38 (8.4%)	43 (9.6%)	47 (10.4%)
**61–70 (n = 406)**	42 (10.2%)	31 (7.6%)	31 (7.6%)	35 (8.5%)	69 (16.8%)
**> 70 (n = 375)**	51 (13.5%)	32 (8.5%)	37 (9.8%)	36 (9.5%)	69 (18.3%)
**p-value (Jonckheere-Terpstra-Test)**	<.05	.43	.71	<.05	<.001

### What is the relationship between childhood adversity, resilience and distress?

**Fig 1:**
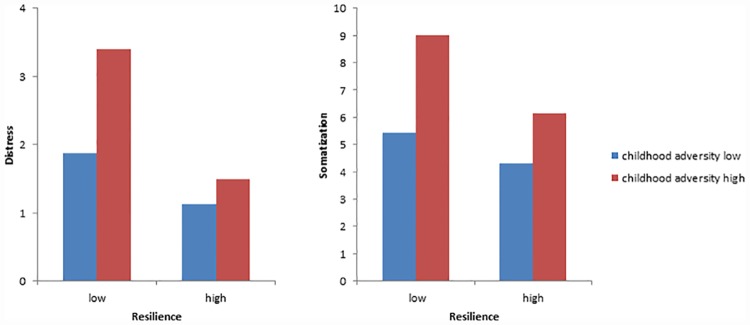
Association between childhood adversity and distress: The buffering effect of resilience.

In order to test our hypothesis that resilience buffers the adverse effects of childhood adversity on distress, we examined the effects of high vs. low childhood adversity and high vs. low resilience on distress and somatic symptoms (Table 3, Fig 1). In two-way ANOVAs all effects were consistent: Distress and somatic symptoms were strongly increased in those who reported significant childhood adversity vs. those who did not; their mean scores were about twice as high. Participants with high resilience had lower symptom scores than those with low resilience. Additionally, as we had hypothesized, there were consistent and strong interactions between childhood adversity and resilience: The highest symptom scores were reported by those participants with high childhood adversity and low resilience. The lowest scores were found for those with low adversity and high resilience. Highly resilient participants had comparatively low scores even in the presence of childhood adversity.

**Table 3:** Association between childhood adversity and distress: The buffering effect of resilience.

*Resilience*						
		low		high	total	
*Distress* ^2)^		Mean (SD)	N	Mean (SD)	N	Mean (SD)
**Childhood adversity**	low^1)^	1.87 (2.30)	1050	1.12 (1.69)	997	1.50 (2.06)
	high	3.40 (2.82)	284	1.50 (2.24)	106	2.88 (2.80)
**total**		2.19 (2.50)	1334	1.15 (1.75)	1103	1.72 (2.25)
***Somatic symptoms***^3)^						
**Childhood adversity**	low	5.42 (5.37)	1061	4.31 (4.63)	1008	4.88 (5.05)
	high	8.99 (6.68)	286	6.14 (5.94)	109	8.21 (6.60)
**total**		6.18 (5.85)	1347	4.49 (4.80)	1117	5.41 (4.47)

Note: CTS = Childhood Trauma Screener; BRCS = Brief Resilience Coping Scale.

^1)^ Cut-off CTS: 0–10 = 0; >10 = 1; cut-off BRCS 0–69 = 0; >69 = 1

Two way ANOVA with CTS and BRCS (df = 1):

^2)^ CTS: F = 97.62; p < .001; BRCS: F = 104.42; p < .001; CTS by BRCS F = 19.18; p < .001

^3)^ CTS: F = 107.14; p < .001; BRCS: F = 38.44; p < .001; CTS by BRCS F = 7.47; p = .006

As childhood adversity and resilience were associated with demographic variables and perceived social support (see Table 4), we determined by testing regression models whether the relationships between childhood adversity, resilience and the dependent variables—distress and somatic symptoms—still remain if we included demographic variables, perceived social support and the experience of unemployment as major stress in adulthood. Table 5 displays that distress was associated with low resilience, low social support, low income, and the lack of a partnership. In addition, distress was positively associated with childhood adversity, age and the experience of unemployment. Somatic symptoms were also predicted by low resilience, low social support, and low income. Positive predictors of somatic symptoms were age, childhood adversity and female sex. Each regression model explained 17% and 24% of variance, respectively.

**Table 4:** Correlations of resilience, childhood adversity, distress, somatic symptoms, perceived social support, and demographic characteristics.

	**1**	**2**	**3**	**4**	**5**	**6**	**7**	**8**	**9**	**10**	**11**	**12**	**13**
**1 Resilience**		-.23**	-.28**	-.28**	-.25**	-.21**	.34**	-.07**	-.05*	-.09**	.15**	.11**	-.05*
**2 Childhood adversity**			.27*	.27**	.24**	.28**	-.37**	.12**	.01	.07**	-.11**	-.16**	.11**
**3 Distress (PHQ4)**				.94**	.94**	.60**	-.25**	.14**	.12**	.12**	-.07**	-.20**	.10**
**4 Depression**					.76**	.56**	-.25**	.14**	.07**	.13**	-.08**	-.19**	.13**
**5 Anxiety**						.56**	-.21**	.12**	.15**	.10**	-.05*	-.18**	.06**
**6 Somatic symptoms**							-.25**	.38**	.12**	.04*	-.11**	-.23**	.02
**7 Social support**								-.11**	.03	-.24**	.05**	.26**	-.13**
**8 Age**									.03	-.12**	-.12**	-.21**	-.08**
**9 Sex**										.08**	-.05**	-.12**	-.04*
**10 Partnership**											-.03	-.34**	.04
**11 Education**												.13**	-.07**
**12 Income**													-.20**
**13 Experienced unemployment**													

Note: Pearson correlation coefficients;

**p < .01;

*p < .05.

**Table 5:** Prediction of distress (PHQ 4) and somatic symptoms (GBB).

	Distress			Somatic symptoms		
Variable	*βeta*	*T*	sign	*βeta*	*T*	sign
**Age**	.11	5.44	.000	.33	17.08	.000
**Sex**	.04	1.87	.062	.08	4.54	.000
**Partnership**	.06	2.81	.005	-.01	-0.31	.758
**Household income**	-.05	-2.20	.028	-.08	-3.72	.000
**Experience of unemployment**	.09	4.60	.000	.03	1.69	.091
**Social Support**	-.08	-3.76	.000	-.11	-5.40	.000
**Childhood adversity**	.17	8.51	.000	.15	7.69	.000
**Resilience (range 0–100)**	-.20	-9.99	.000	-.11	-5.65	.000
**Adj R**^**2**^	.17	F(8,2327) = 59.99	.000	.24	F(8,2353) = 92.70	.000

Note: Sex (1 = male, 2 = female), Partnership (1 = yes, 2 = no), Household income (0 = less than 2000 Euro, 1 = 2000 Euro or more), Experience of unemployment (0 = no, 1 = yes); Variables not in the equation: education.

## Discussion

In a large and representative adult community study, we determined the long-term effects of reported childhood adversities and resilient coping on depression, anxiety and somatic symptoms. Based on a standardized screening, a substantial proportion of 16.2% of the population fulfilled the criteria for significant adverse childhood experiences of child emotional, physical or sexual abuse, respectively emotional and physical neglect. As postulated in our first hypothesis, adverse childhood experiences is linked to heightened vulnerability, such as low resilient coping ability, in terms of helplessness and low self-efficacy. Subjects who reported childhood adversities perceived lower social support over the lifespan, which may be considered as a social component of resilience (see [27]). Thus, dealing with demands and challenges of life proves to be more difficult. Helplessness and low self-efficacy aggravate adaptive coping with conflicts, e.g. in terms of seeking help and developing functional internal beliefs. This, in turn, may lead to more depression, anxiety and somatic symptoms as frequent adult sequelae of childhood adversity in adulthood [53, 54].

Interestingly, significant childhood adversity was also associated with various indicators of lower social status and integration, a lower rate of partnership, less education, income and more experiences of unemployment. Higher age was an additional covariating factor. It should be noted that the oldest cohort had sustained expulsion and family disruption around the 2nd World War. The finding that older people reported more childhood adversity may indicate a cohort effect. As revealed by item specific analysis, both neglect facets were most frequent among the oldest cohort. Alternatively, as child abuse and neglect frequently co-occurs with social deprivation and environmental stress [54], social disadvantage may have translated into a lack of education, finding a suitable partnership and becoming professionally successful. In line with meta-analytical results [63], gender specific analyses revealed significant differences with regard to sexual abuse indicating twice as high percentages for female individuals.

Resilient coping assesses a coping pattern based on tenacity, optimism, active problem solving and active extraction of positive growth. In line with other findings (e.g. [42, 55]), resilient participants reported much less depression, anxiety and somatic symptoms than vulnerable participants. They also perceived better social support, which underlines the protective effect of both variables. Similar to previous studies (e.g. [27]) we found that in the average, men and women without childhood adversity slightly reported higher resilience scores. Overall, the underlying study also confirms our second assumption that childhood adversity is associated with distress, comprising symptoms of depression and anxiety, as well as somatic symptoms throughout the lifespan. Clinical research suggests a link between childhood maltreatment and functional and structural changes of the nervous system leading to a heightened predisposition for depression [64]. It is also suspected that early negative life events result in cognitive deficits, which in turn may influence suicidal behavior [65]. As expected, resilience buffered the effects of childhood adversity on distress [49, 55] and somatoform symptoms. Participants who had sustained childhood adversities but who had developed resilient coping reported less mental and health issues over the lifespan. In our study, we also investigated the effects of objective variables, such as demographic characteristics and social support as source of resilience. However, future research should specify internal and social sources of resilience, such as positive emotions, self-esteem and a good quality of life. Although there are a number of studies considering these psychological sources of resilience [66], there is still a lack of systematic research on the empowerment of subjects with childhood adversity experiences.

Following up on social sources of resilience, one starting point could be the effects of parental care as the first social resource for children [67]. Supportive and loving parenting in childhood promotes not only secure attachment relationships during childhood and adolescence, but has also been linked to positive long-term outcomes such as self-reliance, adaptive emotional regulation and mental health in adulthood [68, 69]. In a recent study [70], we could demonstrate that psychotherapy may provide a correctional experience changing attachment patterns. Thus, the quality of early childhood experiences does not fully determine later psychological health—just as negative life events such as losses or illnesses can shake attachment security and even lead to insecurity [71], psychotherapy can enhance attachment security and possibly promote resilience.

### Strengths and limitations

The strength of this study is the use of standardized scales in a large representative community sample. Unlike previous trials (e.g. [72, 73]), we covered the entire age range from 14 to 92 years. As emphasized in the review of Johnson et al. [66], an expansion of the range of populations investigated is necessary to detect buffering factors due to the requirements of statistical power.

However, we assessed adverse childhood experiences and resilience only by questionnaire, and retrospective reports may be prone to memory bias. The accuracy of retrospectively self-reported childhood trauma might be limited by inaccessibility of traumatic life events or false positive or negative responding (e.g. attributing depressed mood to adversity sustained). Yet, there is also solid support for the reliability of the retrospective assessment of adverse childhood experiences [54]. Other studies have found a good correspondence of retrospective and prospective assessment [10]. Since coping strategies vary depending on personal resources and situational circumstances, future studies should try to assess more detailed information regarding childhood adversity, for instance: frequency, involvement of significant others, age when encountering adverse experiences [74].

We used standardized brief screenings of our core variables, but other sources of adverse childhood experiences, such as loss of a parent, witnessing domestic violence have not been included. Our study focused on the buffering effect of resilient coping style with regard to the association of adverse childhood experiences in general and adverse health outcomes. Since we assessed each subtype of childhood maltreatment with only a single item each in our study, future research using a more comprehensive questionnaire with subscales for the assessment of the different types of childhood maltreatment could analyze whether the buffering effect differ depending on the reported childhood adversity. The generalization of the results might be limited, since the initial response rate was only 55%. It is possible, that individuals refusing participation had relevant tendencies in childhood traumatization. Also, it should be noted at this point that the outcome variables were not operationalized as a clinical diagnosis. The PHQ-4 is only a short screening instrument for depression and general anxiety and does not diagnose a mental disorder according to DSM or ICD classification [59]. Overall, our findings provide insight on the influence of resilient coping style on mental health based on sound data. Although resilient coping style represents a dispositional protective factor, it might be amenable to external stimuli which can be useful for focused interventions and treatments. Taking up on this idea, additionally tracking resilience processes will be a very challenging, but extremely fruitful endeavor for research on resilience. A prospective study involving both, trait resilience and state measures of resilience, as moderating variables will foster a better understanding how some individuals are able to successfully adjust after adverse childhood experiences while others develop psychological or physical disorders.

## Supporting information

S1 Data (SAV)
